# Urine β 2-Microglobolin in the Patients with Congenital Heart Disease

**Published:** 2013-06-01

**Authors:** Noor Mohammad Noori, Simin Sadeghi, Iraj Shahramian, Kambiz Keshavarz

**Affiliations:** 1 Pediatric Cardiology, Children and Adolescent Health Research Center, Zahedan University of Medical Sciences, Zahedan, IR Iran; 2 Pediatric Nephrology, Children and Adolescent Health Research Center, Zahedan University of Medical Sciences, Zahedan, IR Iran; 3 Department of Pediatrics, Children and Adolescent Health Research Center, Zabol University of Medical Sciences, Zabol, IR Iran; 4 Department of Pediatrics, Yasuj University of Medical Sciences, Yasuj, IR Iran

**Keywords:** Congenital Heart Disease, Renal Function, Children

## Abstract

**Background:**

This study aimed to evaluate the renal tubular function in the patients with congenital heart disease using β_2_-microglobulin.

**Methods:**

In this case-control study, based on oxymetry, the patients with congenital heart disease were divided into two groups of cyanotic (n=20) and acyanotic (n=20). Congenital heart disease was diagnosed by echocardiography. Healthy individuals within the same age and sex groups were used as controls. Na^+^, β_2_-micro globulin, creatinine (Cr), and β_2_-microglobulin/Cr ratio were measured in random urine samples and the results were compared to the same parameters in the control group using Tukey, One-Way ANOVA, and X^2^ tests.

**Results:**

Based on the study results, urine sodium in the patients with cyanotic heart disease was significantly different from that of the controls (P=0.023). The results also revealed a significant difference between the two groups with congenital heart disease regarding urine β_2_-microglobulin (P=0.045).

In addition, the patients with cyanotic heart disease were significantly different from those with acyanotic heart disease and the controls regarding urine β_2_-micro globulin/Cr ratio (*P*=0.012 and *P*=0.026, respectively).

**Conclusions:**

The results of this study demonstrated that renal tubular dysfunction began in the patients with congenital heart disease, especially in those with cyanotic congenital heart disease. Besides, early diagnosis before cardiac surgery leads to better control of renal tubular disease.

## 1. Background

Congenital Heart Disease (CHD) occurs in 0.5-0.8% of live births. Congenital heart defects have a wide spectrum of severity in infants.The diagnosis in half of patients is established by the first week of age in and by 1 month of age in rests of patients (1).

Parallel to the increased survival, the risk of secondary renal damage has also increased. Therefore, early diagnosis of renal dysfunction can lead to better management and improvement of the life styles of such patients.

By urinary modern technology, minute quantities of urinary substances, such as low molecular weight protein, *β*_2–_microglobulin, Retinol Binding Protein and, N-acetyl-beta-D- glucosaminidase (as predictive markers in renal tubular disease) can also be measured for early detection of renal function in children (2). Excretory differences of urinary contents can suggest the site or the mechanism of renal damage. Moreover, increased urine beta-2-microglobulin is the result of renal exposure to harmful substances which can lead to renal damage (3). Therefore, evaluation of beta-2–microglobulin is one suitable method in predictive renal involvement.

Dimopoulos et al. showed renal dysfunction in adult patients with CHD. They also demonstrated a three-fold increase in mortality of the patients with CHD when Glomerular Filtration Rate (GFR) decreased moderately or severely (4). Furthermore, Inatomi et al. reported that polycytemia was more responsible for cyanotic nephropathy compared to hypoxia, and the severity of polycytemia was not proportional to increased erythropoietin.

Duration of cyanotic disease is a risk factor for glomerular injury. Hyper viscosity following prolonged cyanotic disease causes a decrease in peritubular capillary blood flow that is responsible for proteinuria following increased glomerular hydrostatic pressure. This phenomenon together with pedocyte dysfunction induces proteinuria. Eventually, prolonged proteinuria causes interstitial renal fibrosis which leads to a decrease in GFR and creatinine clearance (5).

Based on the results of the study by Agras et al., nephropathy is one of the complications of CHD, especially the cyanotic form, and tubular damage develops during the first decade of life in the patients with cyanotic heart disease (6).

Akita et al. also demonstrated that in the patients with cyanotic heart disease, nephropathy is marked with renal tubular dysfunction similar to glomerular dysfunction. They also reported that measurement of urine N-acetyl-*β*_2_ -glucosaminidase (NAG) and urine *β*_2_ micro globulin is beneficial for the early diagnosis of either tubular or glomerular dysfunction in the patients with cyanotic heart disease (7).

In another study, Niboshi et al. showed that all types of non­ionic contrast media cause transient Acute Kidney Injury (AKI) in the children with CHD after cardiac catheterization. In case renal tubular function is undamaged on a long-term basis, one should only be alert to contrast medium-encouraged nephropathy, especially in neonates and infants, the patients receiving more than 5mL/kg of the contrast media in a wide range, and the patients with cyanotic heart disease consuming non-ionic contrast media (8).

Bozkurt et al. showed that the plasma level of *β*_2_- micro globulin increased in the patients with Dilated Cardiomyopathy (DCM) and suggested *β*_2_-micro globulin to be a predictive factor in evaluation of the prognosis of heart failure in the patients with DCM (9).

Andreoli said:“Acute kidney injury (AKI) (previously called acute renal failure) is characterized by a reversible increase in the blood concentration of creatinine and nitrogenous waste products and by the inability of the kidney to regulate fluid and electrolyte homeostasis appropriately” (10). AKI, defined as a rapid decline in GFR, is a common problem whose incidence has been increasing highly predominantly in the hospital settings (10).

In another research, Mehta et al. demonstrated that renal dysfunction was detected in a sick newborn by measurement of *β*_2_-micro globulin in urine. In that study, 90% of the sick newborns who had been admitted in neonatal intensive care unit had raised up *β*_2_-micro globulin in urine due to renal tubular dysfunction (11).

Moreover, Andreoli showed that AKI occurred in 3.9/1000 live births and 34.5/1000 newborns who had been admitted in neonatal intensive care units in a developing country were suffering from AKI. AKI was also prevalent among the neonates who had undergone cardiac surgery (10).

Some biomarkers, such as BUN and creatinine, have been used for diagnosis of AKI since several years ago. Therefore, it seems that some new biomarkers are needed for identifying the high risk patients and early diagnosis of AKI (12).

 The present study aims to assess the renal tubular dysfunction in the patients with cyanotic and acyanitic congenital heart disease (CCHD and ACCHD) using *β*_2_-micro globulin as a marker of tubular function for early diagnosis before cardiac surgery.

## 2. Materials and Methods

In this case-control study, 20 patients above 2 years old who suffered from CCHD and ACCHD were randomly selected from the patients referring to Ali-ebn-e-Abitaleb Hospital affiliated to Zahedan University of Medical Sciences, Zahedan, IR Iran from 2008 to 2010. 

All the patients underwent physical examination and their medical history was taken. Chest x-ray, ECG, and echocardiography were also performed for all the patients. 

Based on echocardiography and oxymetry, CHD and cyanotic (*Spo*_2_<85%) or acyanotic (*Spo*_2_ >85%) diseases were diagnosed and classified.

Then, 20 healthy individuals above 2 years of age who were age- and sex- matched with the case groups were considered as the control group.

Nutritional habit, absence of past medical history of known renal disease, and normal hemodynamic conditions (normal BP, no edema, no proteinuria, and no dehydration sign) were similar in the three study groups. The patients with heart failure, those consuming diuretics, anti-inflammatory drugs, or Angiotensin Converting Enzyme Inhibitors (ACEIs), and those with abnormal urinalysis or positive urine culture were excluded from the study.

Cyanotic heart disease group included 8 patients with Tetralogy of Fallot (TOF), 3 with Single Ventricle (SV), 4 with Ventricular Septal Defect and Pulmonary Stenosis (VSD + PS), 3 with isolated critical PS, and 2 patients with Transposition of Great Arteries (TGA).

On the other hand, the acyanotic group contained 15 patients with VSD±PH including 10 Patients with VSD and normal pulmonary artery pressure and 5 patients with VSD and mild pulmonary hypertension, 2 with Atrial Septal Defect (ASD), 1 with aortic stenosis + aortic insufficiency (AS+AI), 1 with AS, and 1 with ASD + PS.

Blood samples were obtained from the study population to measure CBC, BUN, and Cr and morning random urine samples were taken in order to assess Na, K, Ca, Mg, and *β*_2_-micro globulin.

In order to evaluate renal tubular function, urine sodium/creatinine ratio [Na (meq)/Cr (mg)], urine potassium/creatinine ratio [K (meq)/Cr (mg)], calcium/creatinine [Ca (meq)/Cr (mg)], urine magnesium/creatinine ratio [Mg/Cr], and urine β_2_-micro globuline/creatinine ratio [β2 (mg)/Cr (mg)] were calculated and statistically analyzed.

Urine Naand K, were measured by Felin photometric method,urine Ca was measured with a manual method using Darman-Kav kit, and urine Mg was measured with a manual method using a kit (Zist chimie Co, Tehran, Iran) according to manufacturer’s instructions.

Moreover, urine Cr was measured through RA-1000 autoanalyzer using Eletex kit, while urine *β*_2_-micro globulin was assessed by Elisa method using AS/KU kit.

*X *^2^, One-Way ANOVA, and TUKEY tests were employed for comparing the cyanotic patients to the acyanotic ones and the controls.

## 3. Results

Overall, 40 patients (20 with CCHD and 20 with ACCHD) and 20 healthy individuals were enrolled into and completed the present case-control study.

There were 9 boys and 11 girls in the cyanotic group, 14 boys and 6 girls in the acyonotic group, and 11 boys and 9 girls in the control group. The mean age of the subjects was 5.8±4.3, 5.6±4.7, and 5.4±4.1 years in the cyanotic, acyanotic, and control groups, respectively. 

The results of *X *^2 ^test revealed no significant difference among the three groups regarding sex and age. One-Way ANOVA was used to compare the cyanotic patients’ age and urine K, Ca, Mg, Cr, Na/Cr ratio, K/Cr ratio, Ca/Cr ratio, and Mg/Cr ratio to those of the acyanotic patients and the controls.

The results showed no statistically significant differences between urine Na,K,Mg,Ca,Na/Cr,K/Cr,Mg/Cr and Ca/Cr among the three groups (Table 1), while a significant difference was found regarding urine Na, *β*_2_-micro globulin, and *β*_2_/Cr ratio (Table 2). Furthermore, 5 patients (25%) in the CCHD group showed increased urine *β*_2_-micro globulin. 

**Table 1.** Comparison of the Study Groups Regarding Na, K, Ca, Mg, and Cr

Group	Total (n=60) Mean± SD	CCHD[Table-fn fn5110] (n=20) Mean ±SD	Control (n=20) Mean ±SD	ACCHD[Table-fn fn5110] (n=20) Mean± SD	*P value*
Urine Na	71.53±20.47	62.80±19.99	79.68±15.07	72.1±22.89	00.03
Urine K	48.21±21.99	47.02±17.50	54.42±30.30	43.20± 14.43	0.26
Urine Ca	7.78±4.38	7.97±4.75	6.70±4.09	8.66± 4.27	0.36
Urine Mg	4.23±2.14	4.45±2.41	4.00±1.88	4.17± 2.17	0.72
Urine Cr	48.72±32.25	43.02±26.04	44.76±24.51	58.40± 42.44	0.26
Urine β_2_	0.79±1.55	1.53±2.50	0.48±0.51	0.37± 0.31	0.03
Na/Cr	3.20±6.28	2.94±5.47	2.57±1.97	9.35±4.11	0.27
K/Cr	2.10±3.41	2.34±4.10	1.65±1.67	4.03± 2.30	0.77
Ca/Cr	0.35±0.86	0.55±1.45	0.19±0.19	0.35± 0.31	0.41
Mg/Cr	0.22±0.67	0.31±0.92	0.12±0.12	0.70± 0.25	0.65
β_2_/Cr	0.01±0.03	0.04±0.05	0.01±0.01	0.008± 0.008	0.008

Abbreviations: ACCHD, A cyanotic Congenital Heart Disease; CCHD, Cyanotic Congenital Heart Disease

**Table 2.** Comparison of the Study Groups Regarding Na-Urine, *β_2_*-Urine, and *β_2_*/Cr

Parameter	Group	Mean	Std. Error	*P value* [Table-fn fn5113]
Na-urine	ACCHD Control			
		9.29	6.19	0.298
	CCHD[Table-fn fn5112] Control	-7.58	6.19	0.0444
		-9.29	6.19	0.298
	ACCHD[Table-fn fn5112] CCHD	-16.88	6.19	0.023
		7.58	6.19	-444
β_2_-urine	ACCHD Control	16.88	6.19	0.023
		-1.15	0.47	0.045
	CCHD Control	-0.10	0.47	0.973
		1.15	0.47	0.045
	ACCHD CCHD	1.05	0.47	0.075
		0.10	0.47	0.973
β_2_/Cr	ACCHD Control	-1.05	0.47	0.075
		-0.03	0.01	0.012
	CCHD Control	-0.0003	0.01	0.955
		0.032	0.01	0.012
	ACCHD CCHD	0.029	0.01	0.026
		0.003	0.01	0.955
		-0.029	0.01	0.026

Abbreviations: ACCHD, A Cyanotic Congenital Heart Disease; CCHD, Cyanotic Congenital Heart Disease

The difference is significant at *P*<0.05 level

TUKEY’s test was used in order to determine the differences among the three groups. As Table 2 depicts, urine Na decreased significantly in the patients with CCHD compared to the controls (*P*=0.023). In addition, urine *β*_2_-micro globulin significantly increased in the patients with CCHD compared to those with ACCHD (*P*=0.045). 

Besides, in comparison to the ACCHD patients and the controls, urine *β*_2_/Cr ratio had significantly increased in the patients with CCHD (*P*=0.012 and *P*=0.026, respectively).

## 4. Discussion

Progressive tubular injury is frequently accompanied by glomerular disease associated with proteinuria. Clinical information showed that high levels of proteinuria before, in addition to after beginning of treatment predict rapid decline in renal function and more marked tubulointerstitial injury. Also,the composition of the abnormal protein excretion has a powerful predictive effect on progression of renal function impairment, perhaps reflecting the greater nonselective glomerular wall damage (13).

Fujimoto et al. reported the prevalence of nephropathy in the patients with CCHD to be about 30% which is quite similar to the results of the current study (14). Moreover, Awad et al. showed that kidney damage would occur following cyanotic heart disease, especially in proximal renal tubules (15). In another study, Efren et al. showed no significant correlation between GFR in the patients with CCHD and ACCHD. Also, they revealed the occurrence of AKI in the patients with CCHD (16). Our study results also showed a significant relationship between kidney injury and CCHD.

The results of the current study revealed a significant decrease in the tubular secretion of urine Na in the patients with CCHD compared to the controls. This was consistent with the results obtained by Passwell et al. (4). They showed that leading cause of the low secretion of sodium into urine is increasedoncotic pressure in the peritubular capillaries following polycythemia, due to an increase in tubular sodium reabsorption (4). Similarly, polycythemia was a common finding in most CHD patients of our study, especially the cyanotic ones and this can explain the difference in urine Na secretion in the study population.

Serum creatinine is used to determine renal function all over the world, but has some limitations for assessing GFR. Up to now, various new biomarkers, such as Neutrophil Gelatinase Associated Lipocalin (NGAL), Kidney Injury Molecule-1(KIM-1), cystatin C, Interlukine-18 (IL-18), N-acetyl-*β*_2-_(D)-glucosaminidase(NAG), and β_2_-microglobulin, have been studied for early and more accurate diagnosis of AKI (12,17).

 Some researches have shown that in the adult patients undergoing cardiac surgery, serum and urine NGAL is a valuable predictor of AKI and is associated with AKI duration and severity (17).

In another study, Andreoli SP showed that intravenous infusion of theophylline in asphyxiated neonates improved renal function and decreased of excretion of β_2_-microglobulin.

Conventional drugs used for prevent AKI such as diuretics and 'renal-dose' dopamine and fenoldopam (as a potent short acting selective, dopamine-1receptor agonist) are also believed to prevent AKI (10).

In future several drugs are known to prevent yet to come deterioration of renal function:such as antioxiident,anti-adhesion molecule therapy and the administration of vascular mediators and multipotent mesenchymal stem cells (10).

The increased urinary *β*_2_-microglobulin secretion and random urine *β*_2_/Cr ratio in our CCHD patients compared with the ACCHD ones and the controls demonstrated that tubular dysfunction led to an increase in urine *β*_2_-microglobulin following a tubular reabsorptive disturbance. This finding is comparable to the results of most other researchers.

## 5. Conclusions

The results of the current study showed that renal tubular function was affected in the patients with CHD. Thus, early diagnosis of tubular dysfunction is essential for a better management of these patients before cardiac surgery and measurement of *β*_2_-microglobulin is suggested for an early diagnosis of tubular dysfunction in the patients with CHD.
